# Modified Qing-Zao-Jiu-Fei decoction attenuated pulmonary fibrosis induced by bleomycin in rats via modulating Nrf2/NF-κB and MAPKs pathways

**DOI:** 10.1186/s13020-024-00882-5

**Published:** 2024-01-16

**Authors:** Jia-Qian Zhu, Yuan-Yang Tian, Kam Leung Chan, Zhen Hu, Qing-Qing Xu, Zhi-Xiu Lin, Yan-Fang Xian

**Affiliations:** 1 School of Chinese Medicine, Faculty of Medicine, The Chinese University of Hong Kong, Shatin, N.T., Hong Kong, SAR People’s Republic of China; 2 Hong Kong Institute of Integrative Medicine, The Chinese University of Hong Kong, Shatin, N.T., Hong Kong, SAR People’s Republic of China; 3 Li Dak Sum Yip Yio Chin R&D Centre for Chinese Medicine, The Chinese University of Hong Kong, Shatin, N.T., Hong Kong, SAR People’s Republic of China

**Keywords:** Modified Qing-Zao-Jiu-Fei Decoction (M-QZJFD), Bleomycin, Pulmonary fibrosis, Oxidative stress, NF-κB/Nrf2 pathway, MAPKs pathway

## Abstract

**Background:**

Qing-Zao-Jiu-Fei Decoction (QZJFD) is a famous herbal formula commonly prescribed for the treatment of lung-related diseases in the ancient and modern times. *Trichosanthis Fructus* (TF) and *Fritillariae Thunbergii Bulbus* (FTB) are widely used for treatment of cough and pulmonary disease. In order to identify a more effective formula for treatment of pulmonary fibrosis, we intend to add TF and FTB in QZJFD to form a modified QZJFD (MQZJFD). In this study, we aims to explore MQZJFD as an innovative therapeutic agent for pulmonary fibrosis using bleomycin (BLM)-treated rats and to unravel the underlying molecular mechanisms.

**Methods:**

BLM was given to SD rats by intra-tracheal administration of a single dose of BLM (5 mg/kg). QZJFD (3 g/kg) and MQZJFD (1, 2 and 4 g/kg) was given intragastrically daily to rats for 14 days (from day 15 to 28) after BLM administration for 14 consecutive days.

**Results:**

MQZJFD was found to contain 0.29% of amygdalin, 0.020% of lutin, 0.077% of glycyrrhizic acid and 0.047% of chlorogenic acid. BLM treatment could induce collagen deposition in the lung tissues of rats, indicating that the pulmonary fibrosis rat model had been successfully established. MQZJFD have better effects than the original QZJFD in reducing the pulmonary structure damage and collagen deposition of rat lung fibrosis induced by BLM. MQZJFD could reduce the hydroxyproline content in lung tissues of BLM-treated rats. The biomarkers of fibrosis such as matrix metalloproteinase 9 (MMP9), collagen I and α-smooth muscle actin (α-SMA) were remarkably reduced after treatment with MQZJFD. MQZJFD also have anti-oxidant stress effects by inhibiting the level of malondialdehyde (MDA), but enhancing the activities of superoxide dismutase (SOD) and glutathione peroxidase (GSH-Px), and the level of glutathione (GSH) in the lung tissues of BLM-treated rats. Moreover, the MQZJFD markedly suppressed the over expressions of p-p65/p65 and p-IκBα/IκBα, but upregulated the Nrf2. MQZJFD also suppressed the protein expressions of p-ERK1/2/ERK1/2, p-p38/p38 and p-JNK/JNK in the lung tissues of BLM-treated rats.

**Conclusions:**

MQZJFD could improve the pulmonary fibrosis induced by BLM in rats via inhibiting the fibrosis and oxidative stress via suppressing the activation of NF-κB/Nrf2 and MAPKs pathways.

## Introduction

Idiopathic pulmonary fibrosis (IPF) is a progressive, and irreversible fibrotic lung disease. IPF is usually with a poor prognosis, with inflammation and oxidant stress at the early stage. However, there are no effective drugs in clinic. The most commonly used corticosteroids are broad spectrum anti-inflammatory and anti-fibrotic agents, which always arouse severe side effects such as tuberculosis infection, osteonecrosis and gastrointestinal symptoms after long-term clinical use [1–3]. Therefore, there is a huge unmet need for safe and effective alternative treatments for IPF.

As a form of traditional medical system that has been in practice for thousands of years in China, Chinese medicine has developed medical theories and accumulated a great deal of valuable experience in the prevention and treatment of lung-related diseases [4]. Given that the multiple factors are involved in the pathogenesis of IPF, drugs with multi-target ability are now perceived as a more promising therapeutic strategy for IPF. Chinese medicines have attracted great attention as potential therapeutic agents for IPF in recent years, since they have the characteristics of multiple components, multi-targets and multi-pharmacology.

Qing-Zao-Jiu-Fei Decoction (QZJFD) is a famous herbal formula commonly prescribed for the treatment of lung-related diseases in the ancient and modern times [5–7]. First recorded in the Synopsis of the Golden Chamber (Yi-Men-Fa-Lǜ in Chinese) which was written by the famous Chinese medicine physician Yu Chang in the early Qing dynasty, QZJFD is composed of 9 herbs including *Mori Folium* (Sangye in Chinese), *Gypsum Fibrosum* (Shigao in Chinese), *Ophiopogyonis Radix* (Maidong in Chinese), *Armeniacae Semen Amarum* (Kuxingren in Chinese), *Eriobotryae Folium* (Pipaye in Chinese), *Ginseng Radix et Rhizoma* (Renshen in Chinese),, *Sesami Nigrum Semen* (Heizhima in Chinese), *Asini Corii Colla* (Ejiao in Chinese), *Glycyrrhizae Radix et Rhizoma* (Gancao in Chinese) at the ratio of 3:3:2:2:1:1:1:1:1. In Chinese medicine practice, QZJFD is known to possess therapeutic effects of Clearing dryness and moistening lung, nourishing Yin and tonifying Qi, and is commonly prescribed to treat lung-related diseases, such as cough, radiation-induced liver injury, pulmonary fibrosis, tuberculosis and lung cancer [8–11]. QZJFD has been found to improve acute radiation pneumonia and the incidence of pulmonary fibrosis in patients with advanced lung tumor via reducing the levels of tumor necrosis factor-α (TNF-α), endothelin (ET), connective tissue growth factor (CTGF) and platelet-derived growth factor (PDGF) in plasma [5, 12]. Moreover, a recent study revealed that 70% aqueous ethanol extract of QZJFD significantly alleviated lung injury, decreased the levels of hydroxyproline, transforming growth factor β1 (TGF-β1), TNF-α and interleukin 1 β (IL-1β), but increased the levels of IL-10 and interferon (IFN-γ) in the lung tissues of silica-treated rats, indicating the potential application of QZJFD for clinical treatment of silica-induced pulmonary inflammation and fibrosis [13].

In Chinese medicine practice, *Trichosanthis Fructus* (TF) (Gualou in Chinese) is widely used for treatment of cough and pulmonary diseases [11]. *Fritillariae Thunbergii Bulbus* (FTB) (Zhebeimu in Chinese) has been widely used as an antitussive herb for thousands of years in China [14]. Recent studies revealed that FTB exerted various pharmacological activities such as antitussive, tracheobronchial relaxation, anti-muscarinic, expectorant and pain suppression [15–18]. TF and FTB are also the major components of the some famous Chinese medicine (TCM) formulae such as Qing-Jin Hua-Tan Decoction and Louqin Zhisou Decoction [19, 20]. In order to identify a more effective formula for treatment of IPF, we add TF and FTB in QZJFD to form a modified QZJFD (MQZJFD). In this study, we aimed to investigate this MQZJFD as an innovative therapeutic agent for pulmonary fibrosis using bleomycin (BLM)-induced pulmonary fibrosis in rats and to unravel the underlying molecular mechanisms.

## Materials and methods

### Chemicals and Chinese herbal materials of MQZJFD and QZJFD

Bleomycin (BLM) and tetrandrine (TD) were purchased from Sigma-Aldrich (St. Louis, MO, USA). All other reagents and chemicals used in this study were of analytical grade.

The crude decoction pieces of *Mori Folium*, *Gypsum Fibrosum*, *Trichosanthis Fructus*, *Ophiopogyonis Radix*, *Armeniacae Semen Amarum*, *Fritillariae Thunbergii Bulbus*, *Eriobotryae Folium*, *Ginseng Radix et Rhizoma*, *Sesami Nigrum Semen*, *Asini Corii Colla*, and *Glycyrrhizae Radix et Rhizoma* were purchased from a reliable Chinese herbal supplier with GMP accreditation, and their identities were authenticated by Prof. Yanfang Xian, a seasoned Pharmacognosist at the School of Chinese Medicine, CUHK, where voucher specimen (20210401-20210411) were deposited according to the guidelines of the Chinese Pharmacopoeia 2020. Authenticated voucher specimens were deposited in the store room of School of Chinese Medicine, CUHK, with the voucher specimen numbers listed in Table 1.

**Table 1:** The voucher specimens and references of the 11 Chinese herbal medicines of MQZJFD

Pharmaceutical name	Botanical name	Voucher specimen	References for neuroprotective effect on lung diseases
*Mori Folium* (Sangye)	*Morus alba* L.	20210401	[57 ]
*Gypsum Fibrosum* (Shigao)	*Gypsum Fibrosuum*	20210402	[58]
*Trichosanthis Fructus* (Gualou)	*Trichosanthes kirilowii* Maxim	20210403	[56]
*Ophiopogyonis Radix* (Maidong)	*Camellia petelotii* (Merr.) Sealy	20210404	[59]
*Armeniacae Semen Amarum* (Kuxingren)	*Prunus armeniaca* L. var. ansu Maxim	20210405	[46]
*Fritillariae Thunbergii Bulbus* (Zhebeimu)	*Fritillaria thunbergii* Miq	20210406	[15–17, 45]
*Eriobotryae Folium* (Pipaye)	*Eriobotrya japonica* (Thunb.) LindI	20210407	[60]
*Ginseng Radix et Rhizoma* (Renshen)	*Panax ginseng* C. A. Mey	20210408	[59]
*Sesami Nigrum Semen* (Heizhima)	*Sesamam indicum* L	20210409	[61]
*Asini Corii Colla* (Ejiao)	*Equus asinus* L	20210410	[62]
*Glycyrrhizae Radix et Rhizoma* (Gancao)	*Glycyrrhiza uralensis* Fisch	20210411	[63]

### Preparations the extracts of MQZJFD and QZJFD

The above 11 herbs were ground to powder or pieces, and mixed in the ratio of 3:3:3:2:2:2:1:1:1:1:1. The mixture of QZJFD and MQZJFD were macerated in tenfold distilled water for 12 h at room temperature, then extracted in boiling water for 1 h. The extracts were filtered, and the residue further extracted twice. All three filtrates were combined, and concentrated in a rotary evaporator under negative pressure. Finally, the freeze-dried powers were obtained using a lyophilizer. The yields of MQZJFD and QZJFD were 33.10% and 31.70%, respectively.

### Quality control of MQZJFD and QZJFD

The quality control of MQZJFD and QZJFD was conducted using high performance liquid chromatography (HPLC). Nucleosil 100 C18 HPLC column (4.6 mm × 250 mm, 5 μm) was used for chromatographic separation. The mobile phase was consisted with acetonitrile (solvent A) and 0.1% phosphoric acid (solvent B). The elution progress was as follow: 5–15% solvent A in 0–5 min, 15–50% solvent A in 5–18 min, 50–95% solvent A in 18–19 min, 95–5% solvent A in 19–20 min, 5–5% solvent A in 20–30 min. The flow rate was at 1 mL/min. The injection volume was 10 μL. The separation was set at room temperature and the detection wavelength at 203 nm for amygdalin, 255 nm for rutin and glycyrrhizic acid, and 330 nm for chlorogenic acid.

The powders of MQZJFD and QZJFD were dissolved in distilled water at 200 mg/mL and then filtered through 0.22 μm filter before injection. Four reference compounds such as amygdalin, rutin, glycyrrhizic acid and chlorogenic acid were dissolved in methanol at the stock concentrations of 1.75 mg/mL, 0.08 mg/mL, 0.5 mg/mL and 0.25 mg/mL, respectively, for preparing the specific concentrations. The contents of amygdalin, rutin, glycyrrhizic acid and chlorogenic acid in MQZJFD and QZJFD were calculated using the related standard curves.

### Animals

Adult male Sprague–Dawley (SD) rats (weighing 220–240 g) were obtained from the Laboratory Animal Services Centre, The Chinese University of Hong Kong (CUHK). The animals were housed in controlled conditions with 12 h light/dark cycle at the room temperature (20–24 °C) and the relative humidity (40–60%). The rats had free access to food and water. The whole study was performed in accordance to the Guide for the Care and Use of Laboratory Animals issued by the National Institutes of Health (NIH Publication No. 85-23, revised 2011). All experimental procedures of the whole study were received the approval of the Animal Experimentation Ethics Committee of CUHK (Ref. No.: 20-272-PCF).

### Establishment of BLM-induced pulmonary fibrosis in rats and drugs treatment

BLM-induced pulmonary fibrosis model was established as previous study with minor revision [21]. Briefly, the SD rats were randomly divided into seven groups of 12 animals each: (a) control, (b) BLM plus vehicle, (c) BLM plus MQZJFDL (1 g/kg), (d) BLM plus MQZJFDM (2 g/kg), (e) BLM plus MQZJFDH (4 g/kg), (f) BLM plus QZJFD (3 g/kg) and (g) BLM plus TD (60 mg/kg, twice/week). Tetrandrine, a lignan alkaloid derived from *Stephaniae Tetrandrae Radix*, has been widely used in the treatment of silicosis (also name pneumoconiosis) in China for a long time [22]. It had been confirmed to decrease the size of pulmonary nodules in silicosis mouse models [23]. It also possessed anti-inflammatory, anti-fibrotic, and antioxidant properties [24]. Therefore, In this study, we used tetrandrine as a positive control. BLM was dissolved in physiological saline. BLM was given to rats by intra-tracheal administration of a single dose of 5 mg/kg of BLM (0.25 mL/rat). Rats in control group were given the same volume of physiological saline for the same duration. MQZJFD, QZJFD and TD were dissolved in physical saline and given intragastrically daily to rats for 14 consecutive days (from day 15 to 28) after BLM administration for 2 weeks. The body weight of the rats was monitored and recorded every other day during the whole experiment.

### Histological analysis

Lung tissues were fixed in 10% phosphate-buffered formalin for two days. Subsequently, the tissues were dehydrated using different concentrations of ethanol and embedded in paraffin. The tissues were cut into 6 μm sections. The sections were used for hematoxylin & eosin (H & E) staining and Masson’s trichrome staining as previously described [25]. Briefly, for H & E staining, the sections were incubated with hematoxylin for 5 min following by staining with eosin for 5 min after dewaxing. For Masson’s trichrome staining, the tissue slides were firstly incubated with Bouin’s solution at 56 °C for 15 min after dewaxing. Slides were then stained with hematoxylin for 5 min, and then incubated with Beibrich scarlet-acid fuchsin for 5 min. After staining with hematoxylin and eosin, or Bouin’s solution, all sections were dehydrated with different concentrations of ethanol (from 70 to 100%), and then cleared with xylene and mount. Finally, all slides were observed using a light microscope (Nikon, Japan) after mounting with medium.

### Determination of the total protein level in bronchoalveolar lavage fluid (BALF) of BLM-treated rats

The chest of rats were opened and trachea was exposed under anesthesia, at the end of drugs treatment. The right lung was tied, and the left lung was doused with 300 μL of sterile saline solution to obtain BALF. BALF was centrifuged at 12,000 rpm at 4 °C for 15 min, and the supernatant was collected. The concentration of total protein was determined using BCA assay kit (23225, Thermo).

### Determination of the hydroxyproline content in lung tissues of BLM-treated rats

The hydroxyproline level in the lung tissues of rats were measured using the hydroxyproline assay kit obtained from Abcam (Ab222941). Briefly, lung issues (100 mg) were homogenized in 100 μL of dH_2_O. Then, 100 μL of sample homogenate was transferred to a new screw-capped tube, and added 100 μL of NaOH (10 M) to the homogenate and incubated at 120 °C for 1 h. All samples were cooled down on ice before adding 100 μL of HCl. Finally, the tubes were centrifuged at 10,000 ×*g* for 5 min. Hydroxyproline level in the supernatant was detected as the manufacturer’s instructions.

### Determination of the levels of inflammation cytokines in lung tissues of BLM-treated rats

The levels of TNF-α (ab100785), IL-6 (ab100772), IFN-γ (ab113349) and TGF-β1 (ab119558) in the lung tissues of rats were measured using ELISA kits bought from Abcam as per the manufacturer’s instructions.

### Determination of the expression of the inflammation and fibrosis biomarkers in the lung tissues of rats by immunohistochemistry (IHC)

Paraffin lung tissue sections (6 μm thick) were dewaxed and incubated in citrate buffer (10 mM, pH 6.0) for 20 min at 95 °C. Sections were washed with phosphate buffer solution (PBS) for 5 min for 3 times, then blocked with 1% bovine serum albumin (BSA) in PBST for 30 min. After that, the sections were incubated with the primary antibodies against matrix metalloproteinase 9 (MMP9) (Invitrogen, Cat #MA5-32705), collagen I (Invitrogen, Cat #PA1-26204) and α-smooth muscle actin (α-SMA) (Invitrogen, Catalog #14-9760-82) at 4 °C overnight. After washed with PBS for 5 min for 3 times, the sections were then incubated with the secondary antibodies Alexa Fluor 488 Dunk anti-Rabbit IgG (Abcam, ab150073) or Alexa Fluor 488 Rabbit anti-Mouse IgG (Abcam, ab6728) at room temperature for 1 h. Finally, the sections were mounted using SlowFade Diamond Antifade Mountant with DAPI (Invitrogen, S36973). The images were observed with an image acquisition system (Nikon Instruments Inc. Melville, NY, USA) using a Nikon fluorescent inverted microscope.

### Measurement of the anti-oxidant effects of MQZJFD in the serum of rats

The activities of superoxide dismutase (SOD) and catalase (CAT), and the levels of glutathione (GSH) and malondialdehyde (MDA) in the lung tissues of rats were determined using assay kits. The activity of glutathione peroxidase (GSH-Px) in the serum of rats was assessed using assay kits.

### Real time-polymerase chain reaction (RT-PCR) analysis

Total RNA of lung tissues were extracted using TRIzol (Invitrogen, NY, USA). The concentration of the extracted RNA was determined at 260 nm wavelength. The ratio of absorbance at 260 and 280 nm was used to measure the quality of the RNA. The ratio of A260/A280 was acceptable from 1.9 to 2.1. A mount of total RNA (1.5 μg) was used to synthesize cDNA using TAKARA RR036 PrimeScript RT Master Mix. The cDNA for TNF-α, IL-4, IL-6, IL-1β, TGF-β1, IFN-γ and β-actin were amplified by RT-PCR using TAKARA RR420 SYBR Premix EX Taq with gene specific primers (ATCG Limited) (Primer sequences shown in Table 2). The expression levels of the target genes were calculated using the relative quantification method (2^−ΔΔCT^ method).

**Table 2:** Sequences of primers used for RT- PCR

Gene	Primer sequences (5′-3′)	
IL-1β	Forward	AATCTCACAGCAGCATCTCGACAAG
	Reverse	TCCACGGGCAAGACATAGGTAGC
IL-4	Forward	CAAGGAACACCACGGAGAACGAG
	Reverse	TTCTTCAAGCACGGAGGTACATCAC
IL-6	Forward	ACTTCCAGCCAGTTGCCTTCTTG
	Reverse	TGGTCTGTTGTGGGTGGTATCCTC
TNF-α	Forward	ATGGGCTCCCTCTCATCAGTTCC
	Reverse	CCTCCGCTTGGTGGTTTGCTAC
TGF-β1	Forward	TCCCAAACGTCGAGGTGACC
	Reverse	TGGAGCTGTGCAGGTGTTGA
IFN-γ	Forward	ACAACCCACAGATCCAGCACAAAG
	Reverse	CACCGACTCCTTTTCCGCTTCC
β-actin	Forward	GACAGGATGCAGAAGGAGATTACTG
	Reverse	CCACCGATCCACACAGAGTACTT

### Western blot analysis

Protein of lung tissues was extracted using total protein and nuclear extraction kit obtained from Abcam (ab113474) to get the total and nuclear protein samples. The quantity of protein was measured using BCA assay kit (Thermo, 23225) and then degenerated. Total amount of 15 μg protein were separated by sodium dodecyl sulphate–polyacrylamide gel electrophoresis (SDS-PAGE), then transferred to polyvinylidene fluoride (PVDF) membranes. After blocking with 5% non-fat milk for 1 h, the membranes were then incubated with the primary antibodies against NF-κB p65 (CST, #8242), p-IκBα (CST, #2859), Nrf2 (Proteintech, 16369), collagen I (Abcam, 270993), HO-1 (SCT, 82206), extracellular signal-regulated kinase (ERK1/2), p-ERK1/2, p-p38, p38, c-Jun N-terminal kinase (JNK), p-JNK, p-c-Jun, c-Jun, Lamin B1 (CST, #13435), and GAPDH (Abcam, ab181602) at 1:1000 in 5% BSA of tris buffered saline with tween (TBST) at 4 ℃ overnight. Consequently, the membrances were incubated with relative HRP-conjugated secondary antibodies for 1 h at room temperature, after washing with TBST for 3 times. After that, the bands were exposed using ECL reagent (Thermo, 34580), and the chemiluminescent signals were detected using Azure c300 Chemiluminescent Western Blot Imaging System. The band intensity was quantified using ImageJ software (NIH, USA).

### Acute toxicity study of MQZJFD

Eight-week-old male and female ICR mice were randomly divided into six groups of 20 mice of both sexes (1:1). They were fasted overnight (12 h) with free access only to water prior to administration single doses of MQZJFD (8, 16, 32, 48 and 64 g/kg), which was dissolved in physiological saline. Treatment was given by gavage with the volume of 10 mL/kg. The general behaviors of the mice were continuously monitored for 4 h after the treatment, and intermittent observation was conducted every 6 h during the period of 24 h. After that, the daily observation was lasted for 7 days. The median lethal dose (LD_50_) was calculated as our previous studies [25, 26].

### Statistical analysis

Data was expressed as mean ± standard error mean (SEM). Group differences were analyzed using one-way ANOVA followed by post-hoc Bonferroni test to detect the intergroup differences. Statistical analysis will be performed in GraphPad Prism (Version 9, GraphPad Software, Inc., CA, USA). A difference was considered statistically significant if the *p* value was less than 0.05.

## Results

### HPLC analysis of MQZJFD and QZJFD

**Fig. 1:**
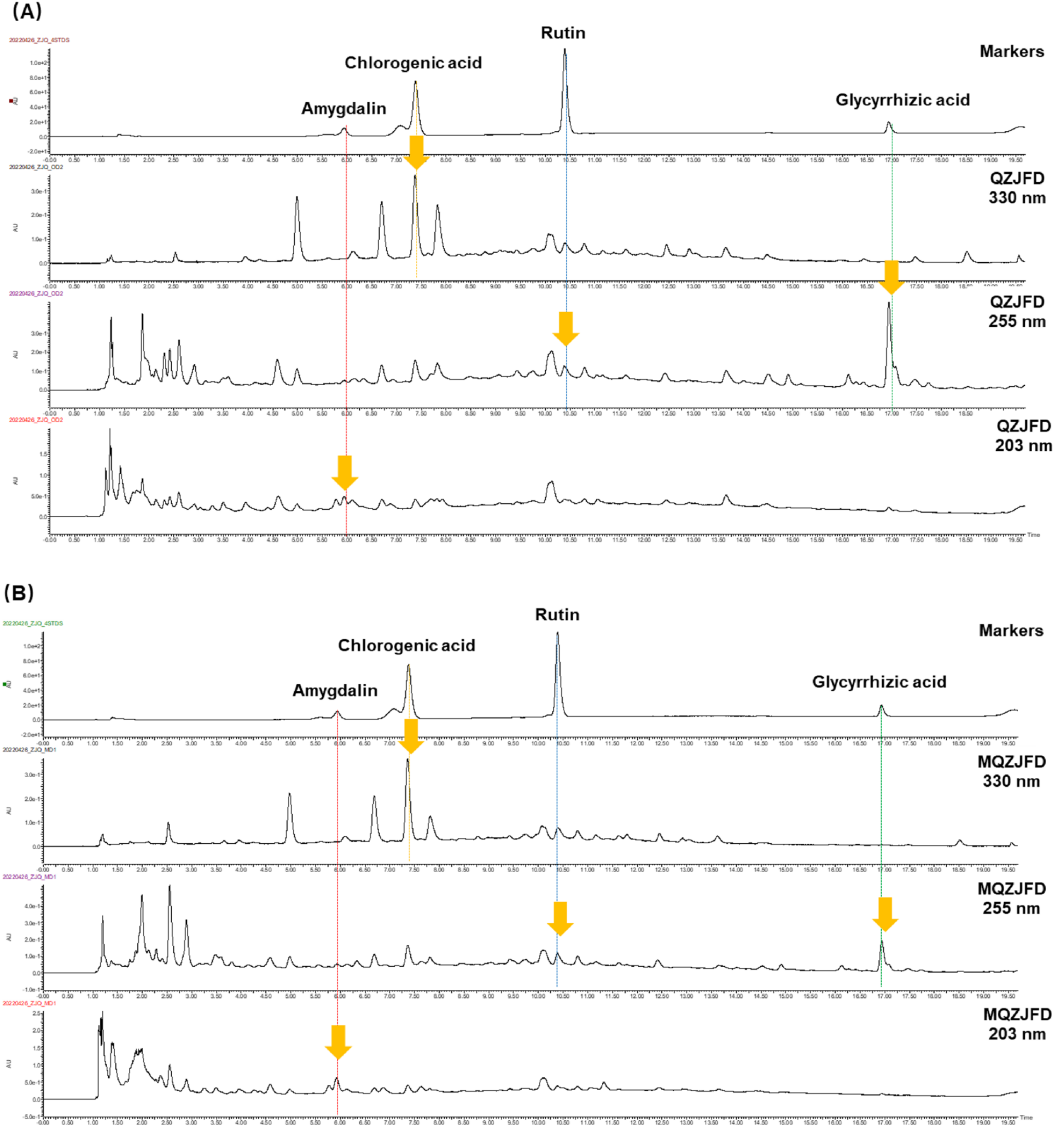
The HPLC profiles of QZJFD, MQZJFD and four reference compounds including amygdalin, rutin, glycyrrhizic acid and chlorogenic acid

HPLC profiles of MQZJFD and QZJFD were shown in Fig. 1. The contents of four compounds such as amygdalin, rutin, glycyrrhizic acid and chlorogenic acid in MQZJFD and QZJFD were calculated using the pre-constructed standard curves shown in Table 3. MQZJFD was found to contain 0.29% of amygdalin, 0.020% of rutin, 0.077% of glycyrrhizic acid and 0.047% of chlorogenic acid. The QZJFD was found to contain 0.161% of amygdalin, 0.030% of rutin, 0.153% of glycyrrhizic acid and 0.049% of chlorogenic acid.

**Table 3:** Linearity range of 4 compounds in HPLC analysis of MQZJFD and QZJFD

Compounds	Linearity		
	Range (μg/mL)	Equation	R^2^
Amygdalin	87.5–1750.0	y = 113.53x−505.44	0.9985
Rutin	4.17–83.33	y = 306.7x−418.41	0.9965
Glycyrrhizic acid	25.0–500.0	y = 139.86x−1270	0.9931
Chlorogenic acid	50.0–250.0	y = 428.54x−4852.5	0.9989

### Effect of MQZJFD on hydroxyproline content, the histopathological examinations and collagen deposition of BLM-treated rats

**Fig. 2:**
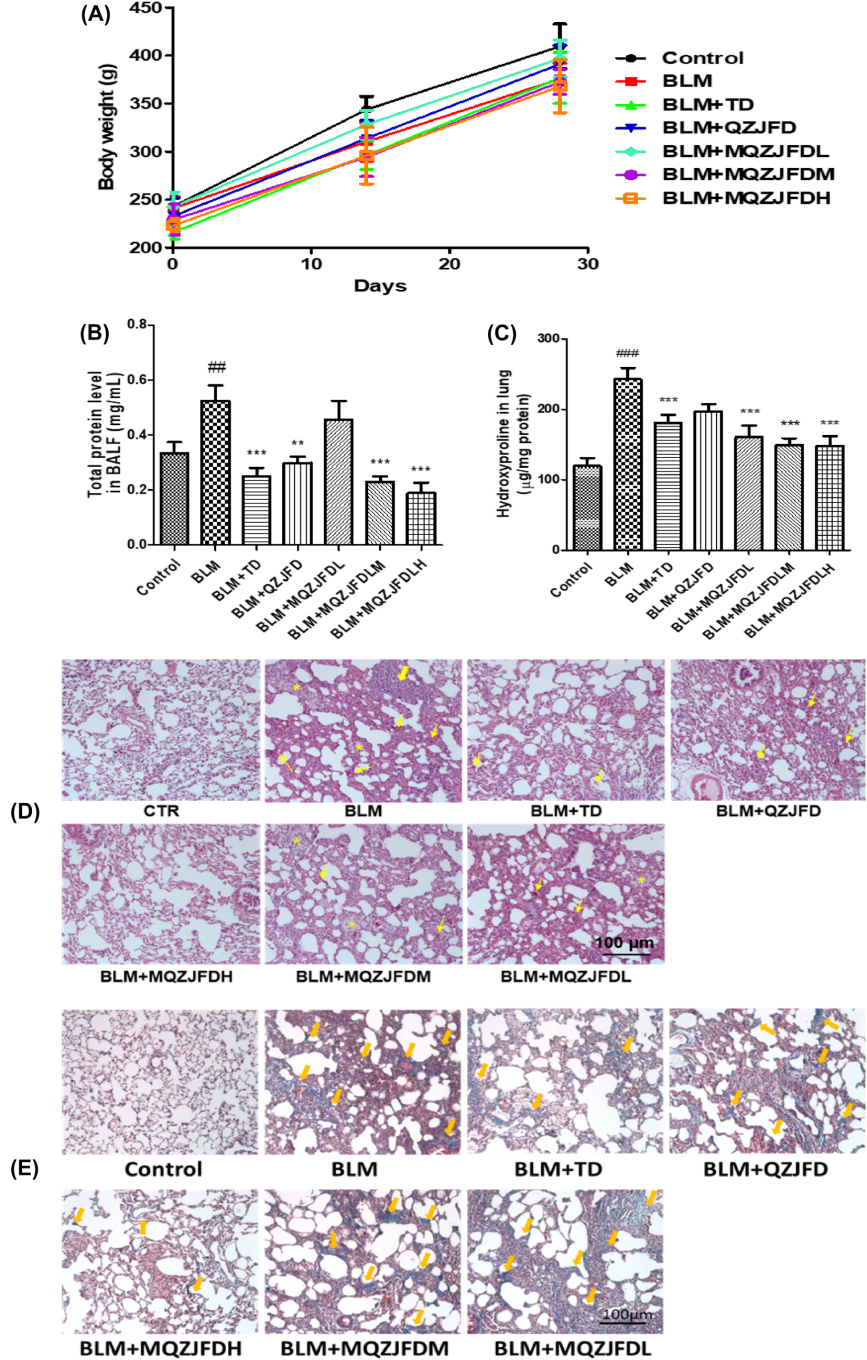
Effects of MQZJFD on the histopathological examinations and collagen examinations on the lung tissues of BLM-treated rats. **A** Body weight of the rats in the BLM-treated rats. **B** The total protein level in BALF of BLM-treated rats. **C** The hydroxyproline content in lung tissues of BLM-treated rats. **D** The histopathological examinations of the lung tissues of BLM-treated rats were detected using H & E staining. **E** The collagen expression of the lung tissues of BLM-treated rats were examined using Masson’s trichrome staining. Magnification at 100 μm. Data were presented as mean ± SEM (n = 4–12). ^##^*p* < 0.01 and ^###^*p* < 0.001 compared to the control group; **p < 0.01 and ***p < 0.001 compared to the BLM group

As shown in Fig. 2A, before and after treatment with BLM, QZJFD or MQZJFD, the body weight of rats did not show any significant difference in all groups on day 0, 14 and 28. In addition, the results of acute toxicity study indicated that MQZJFD at up to the dose of 64 g/kg, which was the maximum tolerable dose of MQZJFD in mice, did not exhibit any observable toxicity.

As shown in Fig. 2B**,** total protein level in BALF was significantly increased (*p* < 0.01) in BLM-treated group, as compared to the control group. However, the total protein level in BALF was markedly reduced after treatments with TD (*p* < 0.05), QZJFD (*p* < 0.001), MQZJFDM (*p* < 0.001) or MQZJFDH (*p* < 0.001) for 2 weeks, as compared with the BLM-treated control group.

As shown in Fig. 2C**,** the hydroxyproline level was significantly increased (*p* < 0.001) in lung tissues of BLM-treated group, as compared to the control group. However, the level of hydroxyproline was markedly reduced after treatment with TD (*p* < 0.001), MQZJFDL (*p* < 0.001), MQZJFDM (*p* < 0.001) or MQZJFDH (*p* < 0.001) for 2 weeks, as compared with the BLM-treated control group. QZJFD treatment did not affect the hydroxyproline level in lung tissues of BLM-treated rats, as compared with the BLM-treated control group.

As shown in Fig. 2D, the lungs of untreated SD rats showed normal alveolar pattern with broncheoli surrounded by alveolar sacs and alveoli separated by alveolar septa. Alveolar hemorrhage was appeared in the lung tissues of BLM-treated rats, but not in control, QZJFD and MQZJFD treatment groups. In addition, hemosiderin deposit and alveolar macrophages were also appeared in the lung tissues of BLM-treated rats. However, treatment with TD, QZJFD or MQZJFD improved the hemosiderin deposit and alveolar macrophages in the lung tissues of BLM-treated rats. Moreover, interstitial inflammation and peribronchial fibrosis could be observed in the lung tissues of BLM-treated rats. All these alternations induced by BLM were reversed by treatment with TD, QZJFD or MQZJFD.

As shown in Fig. 2E, collagen deposition was observed in BLM treated groups. Enlarged alveoli and thickened pulmonary interstitium were also found in the lung tissues of BLM-treated rats. Treated rats with TD, QZJFD or MQZJFD for 2 weeks could alleviate the level of collagen deposition induced by BLM in the lung tissues of rats.

### Effects of MQZJFD on fibrosis biomarkers of BLM-treated rats

**Fig. 3:**
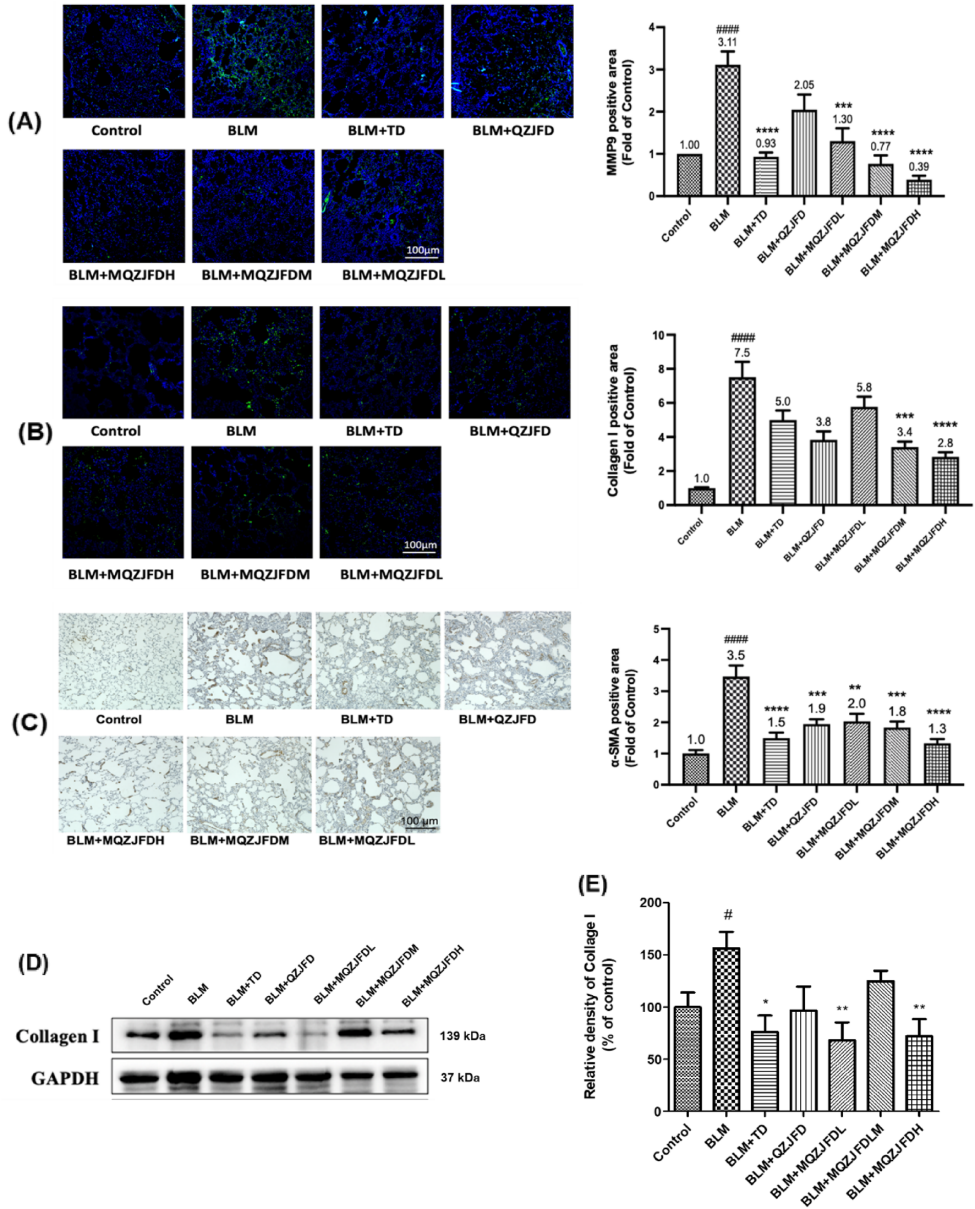
Effects of MQZJFD on the MMP9, collagen I and α-SMA expression in the lung tissues of BLM-treated rats. **A**–**C** Analysis of immunofluorescence staining of MMP9, collagen I and α-SMA expression in the lung tissue of the BLM-treated rats. **D** Representative immunoblot bands for collage I in the lung tissue of the BLM-treated rats. **E** Quantitative analysis of immunoblot bands. Data were presented as mean ± SEM (n = 4). ^#^*p* < 0.05 and ^####^*p* < 0.0001 compared to the control group; *p < 0.05, **p < 0.01, ***p < 0.01 and ****p < 0.0001 compared to the BLM control group. Scale bar: 100 μm

As the IHC images showed in Fig. 3A–C, the protein expressions of MMP9, Collagen I and α-smooth muscle actin (α-SMA) in the lung tissues of BLM-treated rats were significantly increased, as shown with the control group. Treatment with QZJFD could significantly inhibit the protein expressions of α-SMA in the lung tissues of BLM-treated rats, as compared with the BLM control group. In addition, treatment with MQZJFD (1, 2 and 4 g/kg) effectively reduced the protein expressions of MMP9 (*p* < 0.001, *p* < 0.0001 and *p* < 0.0001, respectively), Collagen I (*p* > 0.05, *p* < 0.001 and *p* < 0.0001, respectively) and α-SMA (*p* < 0.01, *p* < 0.001 and *p* < 0.0001, respectively) in a dose-dependent manner, as compared with the BLM control group. Moreover, treatment with TD markedly suppressed the protein expressions of MMP9 and α-SMA (*p* < 0.0001 for both), as compared with the BLM control group. Consistently, as shown in Fig. 3D, E**,** after treatment with BLM, the protein expression of collage I significantly increase in the lung tissues of rats, compared to the control group (2.16-fold). However, treatment with TD, MQZJFDL and MQZJFDH could significantly inhibit the protein expression of collage I (*p < 0.05, **p < 0.01 and **p < 0.01, respectively) in the lung tissues of BLM-treated rats, as compared with the vehicle control group.

### Effects of MQZJFD on oxidant stress in the lung tissues of BLM-treated rats

**Fig. 4:**
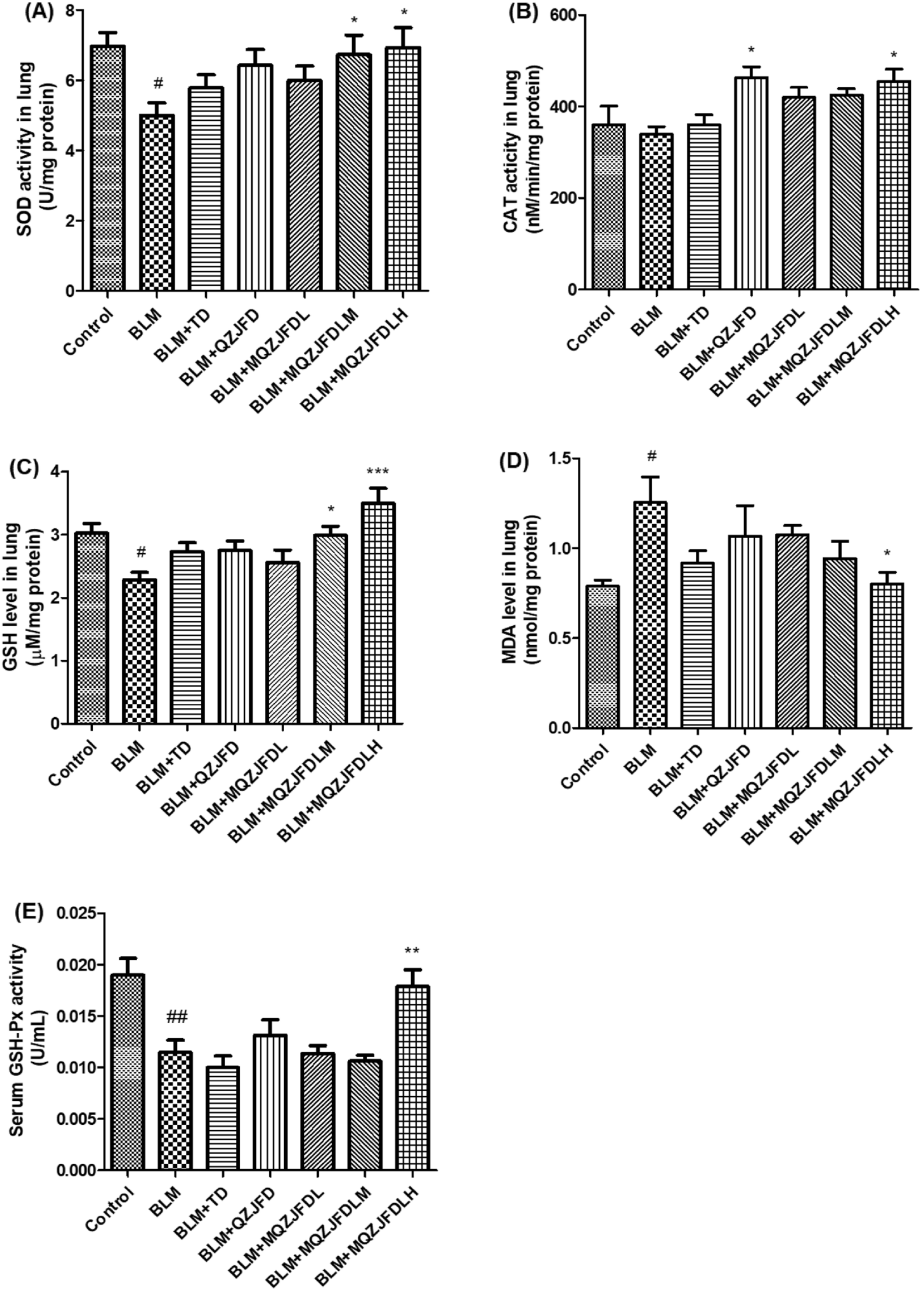
Effects of MQZJFD on oxidant stress in the lung tissues of BLM-treated rats. **A** Serum SOD activity, **B**, **C** MDA and GSH contents, and **D** CAT activity in the lung tissue of BLM-treated rats. **E** Serum GSH-Px activity of BLM-treated rats. Data was presented as mean ± SEM (n = 6). ^#^*p* < 0.05 and ^##^*p* < 0.01 compared to the control group; *p < 0.05, **p < 0.01 and ***p < 0.001 compared to the BLM control group

As the results shown in Fig. 4, as compared with the control group, BLM treatment could significantly decrease the activities of SOD (*p* < 0.05) in lung tissues and GSH-Px (*p* < 0.01) in serum, as well as the GSH level (*p* < 0.05) in lung tissues, while significantly increase the MDA level (*p* < 0.05) in lung tissues of BLM-treated rats. However, treatment with MQZJFDH could markedly enhance the activities of SOD (*p* < 0.05) and CAT (*p* < 0.05), and the level of GSH (*p* < 0.001) in lung tissues and GSH-Px (*p* < 0.001) in the serum of BLM-treated rats, while decrease the MDA level (*p* < 0.05) in lung tissues of BLM-treated rats, as compared with the BLM control group. In addition, treatment with MQZJFDM could significantly enhance the activity of SOD (*p* < 0.05), and GSH level (*p* < 0.05) in lung tissues of BLM-treated rats, as compared with the BLM control group. Treatment with TD also significantly enhanced the activity of CAT (*p* < 0.05) in lung tissues of BLM-treated rats, as compared with the BLM control group.

### Effects of MQZJFD on the inflammation and fibrosis markers in lung tissues of BLM-treated rats

**Fig. 5:**
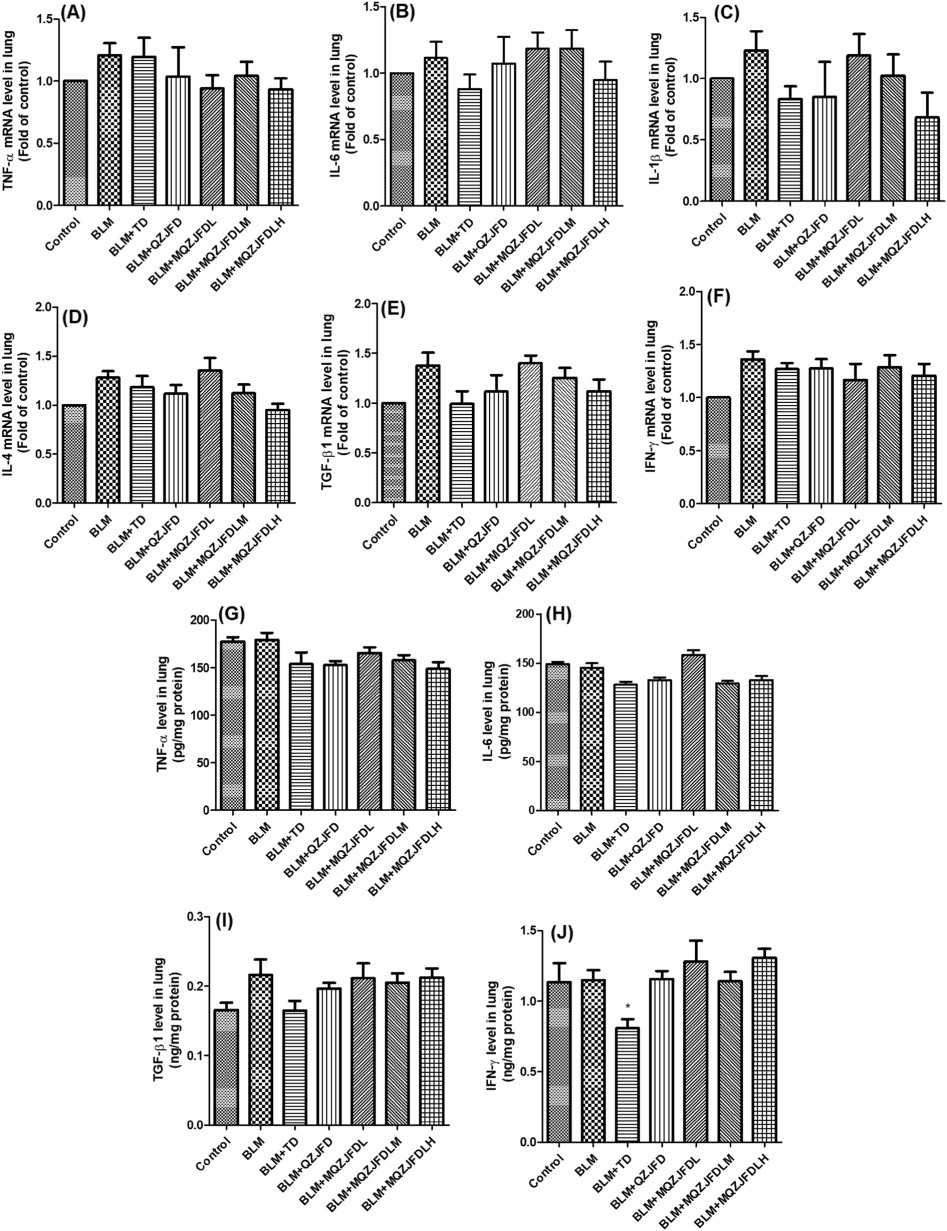
The effects of MQZJFD on the mRNA and protein expression levels of cytokines in lung tissues of BLM-treated rats. **A-F** mRNA levels of cytokines in lung tissues. **G-J** Protein levels of cytokines in lung tissues. Data were presented as mean ± SEM (n = 4–6). *p < 0.05 compared to the BLM control group

As the results shown in Fig. 5A–F, as compared with the control group, the mRNA levels of TNF-α, IL-6, IL-1β, IL-4, TGF-β1 and IFN-γ in lung tissues of rats did not show any significant change in all groups.

As shown in Fig. 5G–J, there was no significant differences in the levels of TNF-α, IL-6 and IFN-γ in lung tissues between the BLM group and control group. TD treatment could significantly reduce the levels of IFN-γ (*p* < 0.05) in the lung tissues of BLM-treated rats, as compare with the BLM control group. However, QZJFD or MQZJFD treatment did not affect the levels of TNF-α, IL-6, IFN-γ and TGF-β1 in lung tissues of BLM-treated rats.

### Effects of MQZJFD on the related protein expressions of Nrf2/NF-κB and MAPKs pathways in the BLM-treated rats

**Fig. 6:**
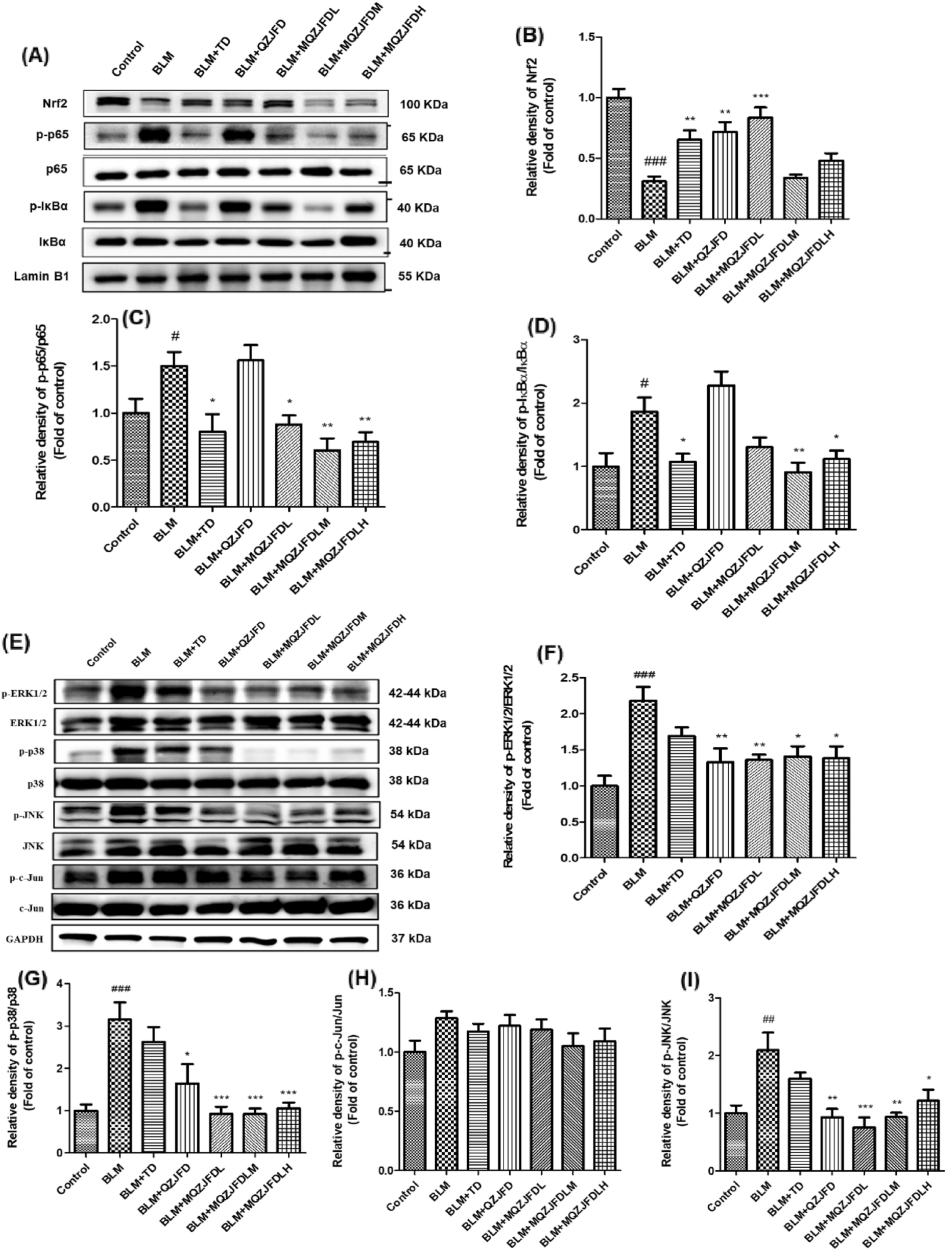
Effects of MQZJFD on the Nrf2/NF-κB pathway in the lung tissue of the BLM-treated rats. **A** Representative immunoblot bands for Nrf2, p-p65, p65, p-IκBα and IκBα in the lung tissue of the BLM-treated rats. **B**–**D** Quantitative analysis of immunoblot bands. **E** Representative immunoblot bands of p-ERK1/2, ERK1/2, p-p38, p38, p-JNK, JNK, p-c-Jun, c-Jun and GAPDH in the lung tissue of the BLM-treated rats. **F**–**I** Quantitative analysis of immunoblot bands data were presented as mean ± SEM (n = 3). ^#^*p* < 0.05, ^##^*p* < 0.01 and ^####^*p* < 0.0001 compared to the control group; *p < 0.05, **p < 0.01 and ***p < 0.001 compared to the BLM control group

As shown in Fig. 6A–D**,** after treatment with BLM, Nrf2 level in the rats showed notable decrease (0.3-fold, *p* < 0.001), as compared to the control group. Accompanied by phospho-IκBα (p-IκBα) in the BLM group, the protein expression of the ratios of p-p65/p65 (1.50-fold, *p* < 0.05) and p-IκBα/IκBα (1.86-fold, *p* < 0.05) in the nucleus also increased remarkably, as compared with the control group. However, after treatment with MQZJFD, the protein expressions of Nrf2, p-p65/p65 and p-IκBα/IκBα were significantly inhibited. Treatment with MQZJFDL significantly increased the protein level of Nrf2 (*p* < 0.001) in lung tissues of BLM-treated rats, as compared with the BLM control group. Treatment with MQZJFDL, MQZJFDM and MQZJFDH markedly decreased the protein expression of the ratio of p-p65/p65 (*p* < 0.05, *p* < 0.01 and *p* < 0.01, respectively), as compared with the BLM control group. The protein expression of p-IκBα/IκBα was also significantly decreased by MQZJFDM and MQZJFDH (*p* < 0.01 and *p* < 0.05, respectively), as compared with the BLM control group.

As shown in Fig. 6E–I**,** after treatment with BLM, the protein expression of p-NF-κB, p-IκBα, p-ERK1/2, p-p38, p-JNK and p-c-Jun were increased, as compared with the control group. However, treatment with MQZJFD (1, 2 and 4 g/kg) could reduce the protein expression of p-ERK1/2/ERK1/2 (*p* < 0.01, *p* < 0.05 and *p* < 0.05, respectively), p-p38/p38 (*p* < 0.001 for all), p-JNK/JNK (*p* < 0.001, *p* < 0.01 and *p* < 0.05, respectively), but not affect the protein expression of p-c-Jun/c-Jun in the lung tissues of BLM-treated rats, as compared with the vehicle control group.

## Discussion

BLM is a therapeutic agent used for treatment of testicular, head and neck cancers, lymphogranuloma and soft tissue sarcomas [27]. However, BLM was found to damage the alveolar lung cells via increasing the collagen tissues in airbags of lung, and inducing fibroblast proliferation [28]. After stimulation with BLM, the inflammation response is usually at the early stage and fibrosis forms at the later stage. Therefore, BLM is widely used to establish pulmonary fibrosis model to investigate the protective effect of agents since it is easy to induce lung fibrosis [29]. In this study, BLM treatment could induce pulmonary inflammation and fibrosis in the lung tissues of rats, indicating that the pulmonary fibrosis rat model had been successfully established, while BLM treatment did not show any changes of the pro-inflammatory cytokines in the lung tissues of rats. MQZJFD has better effects on reducing the pulmonary inflammation and fibrosis than the original QZJFD in BLM-treated rats. MQZJFD have better effects than the original QZJFD in reversing the pulmonary structure damage and collagen deposition of rat lung fibrosis induced by BLM. MQZJFD could reduce the hydroxyproline content in lung tissues of BLM-treated rats. MQZJFD also have anti-oxidant stress by modulating the levels of MDA and GSH, and the activities of SOD and GSH-Px in the lung tissue. The biomarkers of fibrosis, MMP9, Collagen I and a-SMA were remarkably reduced after treatment with different dose of MQZJFD. In addition, MQZJFD also reduced the protein expression of, p-p65/p65 and p-IκBα/IκBα, but increased the expression of Nrf2. Moreover, the over expression of p-ERK1/2/ERK1/2, p-p38/p38, p-JNK/JNK and p-c-Jun/c-Jun in the BLM model can be suppressed by MQZJFD treatment.

Oxidative stress is considered a major pathogenic feature of pulmonary fibrosis. GSH and SOD are the major components of antioxidant defenses systems. It has been demonstrated that elevating the activities of SOD, CAT and GSH-Px, and the level of GSH, but decreasing the level of MDA are helpful to combat against oxidative stress [30]. In this study, BLM could lead oxidative stress via decreasing the level of GSH, the activities of SOD, CAT and GSH-Px, but increasing the level of MDA in the lung tissues of rats, which is consistent with the other studies [31]. Treatment with MQZJFD could suppress the oxidative stress induced by BLM in rats.

Collagen I, a major component of the extracellular matrix in lung tissue, significantly elevated in pulmonary fibrosis [32]. Activation of α-SMA has been considered to play a critical role in pulmonary fibrosis. Higher concentrations of total protein in BALF, the levels of hydroxyproline and expression of α-SMA are concurrent with more collagen deposition, inducing more severe pulmonary fibrosis [33, 34]. It has been revealed that there is a strong relationship between the extent of the fibrosis and the MMP‐9 levels, indicating that MMP‐9 plays a crucial role in the pathogenesis of pulmonary fibrosis [35]. In this study, we used IHC analysis to measure the protein expression of α-SMA, collagen 1 and MMP-9 in the lung tissues of BLM-treated rats. The results showed that the protein expression of α-SMA, collagen 1 and MMP-9 significantly enhanced in the lung tissues of BLM-treated rats, while MQZJFD treatment reversed these changes.

NF-κB was the important transcription factors that were downstream of ROS and MAPKs, and inhibited the activation of NF-κB markedly alleviated the lung injury [36, 37]. NF-κB could promote the profibrotic factors in pulmonary fibrosis. On the other hand, Nrf2 plays a critical role in regulating the antioxidant factors to enable it to further modulate the oxidation/antioxidation capacity [38]. It has been reported that inhibition of Nrf2 could enhance the anti-oxidase defenses system in pulmonary fibrosis [31]. Moreover, NF-κB has been found to modulate the Nrf2 expression or interfere the interactions between Nrf2 and ARE sequences [11]. Therefore, targeting the NF-κB/Nrf2 pathway is a potential novel therapeutic target for pulmonary fibrosis. In this study, we found that MQZJFD could modulating the NF-κB/Nrf2 pathway in the lung tissues of BLM-treated rats, indicating that MQZJFD improve the pulmonary fibrosis induced by BLM in rats may via suppressing the activation of NF-κB/Nrf2 pathway.

Mitogen-activated protein kinases (MAPKs) exist has three major groups including ERK1/2, JNK and p38 [39]. A number of evidence revealed that the activation of JNK and p38 pathways can be activated by oxidative stress [40, 41]. Oxidative stress also provokes the phosphorylation of ERK and regulates the activation of ERK pathway [42]. In addition, NF-κB can be activated by MAPKs pathway in fibrosis [43]. MAPK/NF-kB pathway plays an important role in the pathogenesis of pulmonary fibrosis [44]. In this study, we found that MQZJFD markedly suppressed the protein expression of the phosphorylation of ERK1/2, JNK and p38 in the lung tissues of BLM-treated rats, demonstrating MQZJFD can inhibit the activation of MAPKs pathway.

Although the exact mechanisms of the anti-fibrotic effects of MQZJFD remains to be further elucidated, the multiple components may play critical role in the anti-fibrotic effects of MQZJFD. Ruscogenin, the most active compounds of *Ophiopogyonis Radix*, ameliorates lipopolysaccharides-induced pulmonary endothelial barrier dysfunction by modulating TLR4/Src/VE-cadherin pathway through regulating the interactions of non-muscle myosin heavy chain IIA (NMMHC IIA)-Toll-like receptor 4 (TLR4) [45]. Amygdalin, an active ingredient of *Armeniacae Semen Amarum*, was been found to reduce pulmonary fibrosis induced by BLM in rats via decreasing the type I and III collagen [46]. In addition, amygdalin exerts potential anti-pulmonary fibrosis effects via modulating TGF-β1 signaling in vivo and in vitro [47, 48]. Moreover, amygdalin was also reported to improve the LPS-induced acute lung injury via suppressing the NF-κB and NLRP3 pathways [47, 49]. MQZJFD contain higher content (1.8-fold) of amygdalin than QZJFD (Fig. 1), indicating that adding TF and FTB could increase the content of amygdalin in MQZJFD. Similarly, chlorogenic acid, a typical active components of *Mori Folium*, effectively improve the pulmonary fibrosis induced by BLM in mice via alleviating the expressions of collagen I and α-SMA in dose-dependent manner, and the levels of cleaved caspase-3, caspase-12, and caspase-9, but elevating the level of uncleaved PARP through downregulating the level of phosphorylation of ERK [25]. Chlorogenic acid ameliorates BLM-induced pulmonary fibrosis through modulating autophagy [50]. MQZJFD contain lower content (1.0-fold) of chlorogenic acid than QZJFD (Fig. 1), indicating that adding TF and FTB may reduce the content of chlorogenic acid in MQZJFD. Sesamin, isolated from *Sesami Nigrum Semen*, protected against the airway fibrosis induced by ovalbumin via reducing the activation of myofibroblast and the accumulation of collagen via modulating the TGF-β1/Smad3 pathway [51]. Rutin, another major compound of QZJFD and MQZJFD ameliorated the lung fibrosis induced by BLM in mice through suppressing the TGF-β1/α-SMA/Col I and III pathway [52, 53]. Glycyrrhizic acid was also reported to alleviate BLM-induced pulmonary fibrosis in mice and rats [54, 55]. The different ingredient in different herbs in QZJFD or MQZJFD may interact with each other or act synergistically to exert the therapeutic effects against pulmonary fibrosis.

## Conclusions

In summary, the mechanism of the anti-fibrosis effects of MQZJFD mainly related with modulating NF-κB/Nrf2 and MAPKs pathways in the lung tissues of BLM-treated rats.

## Data Availability

All data supporting the conclusions of this article are included in this article.

## Abbreviations

α-SMA: α-Smooth muscle actin
BALF: Bronchoalveolar lavage fluid
BLM: Bleomycin
CAT: Catalase
CTGF: Connective tissue growth factor
ET: Endothelin
FTB: *Fritillariae Thunbergii Bulbus*
GSH: Glutathione
GSH-Px: Glutathione peroxidase
HPFs: Five high-power fields
IFN-γ: Interferon
IHC: Immunohistochemistry
IL-1β: Interleukin 1β
LD_50_: Median lethal dose
MDA: Malondialdehyde
MQZJFD: Modified QZJFD
NMMHC IIA: Nnon-muscle myosin heavy chain IIA
PDGF: Platelet-derived growth factor
p-IκBα: Phospho-IκBα
QZJFD: Qing-Zao-Jiu-Fei Decoction
SD: Sprague–Dawley
SOD: Superoxide dismutase
TD: Tetrandrine
TF: *Trichosanthis Fructus*
TGF-β1: Transforming growth factor β1
TLR4: Toll-like receptor 4
TNF-α: Tumor necrosis factor-α
